# Soil 16S DNA sequence data and corresponding soil property and wheat yield data from a 72-plot field experiment involving pulses and wheat crops grown in rotations in the semiarid prairie

**DOI:** 10.1016/j.dib.2019.103790

**Published:** 2019-02-28

**Authors:** Chantal Hamel, Yantai Gan, Duaine Messer, Luke D. Bainard

**Affiliations:** a Quebec Research and Development Centre, Agriculture and Agri-Food Canada, 2560 Hochelaga Boulevard, Quebec, QC G1V 2J3, Canada; b Swift Current Research and Development Centre, Agriculture and Agri-Food Canada, 1 Airport Road, Swift Current, SK S9H 3X2, Canada

## Abstract

The soil bacteria diversity and corresponding environmental data made available here are from a 72-field plot experiment testing the effect of pulse frequency in nine wheat-based rotation systems, in the semiarid prairie. The data include sequences of the V6–V8 regions of bacterial 16S rDNA from soil and root extracts, generated using Roche GS FLX Titanium technology, and associated environmental data, specifically levels of soil organic carbon, total carbon, total nitrogen, total phosphorus, pH, electrical conductivity, and extractible sulfate sulfur, copper, iron, manganese, zinc, potassium, nitrate nitrogen, phosphate phosphorus, calcium, and magnesium in the 0–15 cm soil layer, and mineral nitrogen and phosphate in the 0–120 cm soil layer. The grain yield of wheat in the last (4th) phase of the crop rotation systems is also given. The data can be used in meta-analyses of the effect of pea, lentil and chickpea in wheat-based cropping systems on soil bacterial diversity or for monitoring the evolution of soil bacteria communities in cultivated prairie soils in the context of climate change. Samples were collected between 2012 and 2014.

**Table:** Specifications table

Subject area	Soil science, molecular microbial ecology, agronomy.
More specific subject area	Soil bacteria diversity, root-microbe interactions.
Type of data	Tables.
How data were acquired	Sequence data: Amplicon pyrosequencing with Roche GS FLX Titanium technology; UPARSE pipeline [1] to produce OTUs with 97% similarity. Taxonomic assignment with RDP classifier and the 16S rRNA training set 16 [2]. Soil property data: Elemental analyzer (Elementar vario MICRO cube); segmented flow auto-analyzer (Technicon, AAII System, Tarrytown, NY); ICP-OES (Fisher Scientific iCAP6300 Duo); atomic absorption spectrometer (Hitachi Z-8200); pH meter (Fisher Scientific accumet™ AB150); EC meter (Mettler Toledo SevenMulti)
Data format	Analyzed data
Experimental factors	Frequencies of pea (*Pisum sativum* L. cv CDC Meadow), lentil (*Lens culinaris* Medik. cv CDC Maxim), or chickpea (*Cicer arietinum* L. cv CDC Frontier) crops in 4-phase wheat-based crop rotation systems. The wheat cultivar was AC Lillian hard red spring wheat (*Triticum aestivum* L.) in 2010–2013 and Brigade durum wheat (*Triticum turgidum* ssp. *durum*) in 2014
Experimental features	Field experiment with four repetitions in blocks conducted in 2012–2013 and replicated in 2013–2014 on adjacent land in the same field.
Data source location	Swift Current Research and Development Centre of Agriculture and Agri-Food Canada, near Swift Current, Saskatchewan, Canada (50°16′ N, 107° 46′ W).
Data accessibility	Soil and wheat yield data are included with this article. The sequence data have been deposited in NCBI and can be traced using the information in Table 1.
Related research article	C. Hamel, Y. Gan, S. Sokolski, L. Bainard. High frequency cropping of pulses modifies soil nitrogen level and the rhizosphere bacterial microbiome in 4-year rotation systems of the semiarid prairie. Appl. Soil Ecol. (2018) 126, 47–56.

**Table:** 

**Possible use of the data** • For use in meta-analyses. • Can be used to monitor the evolution of the bacterial communities associated with the roots and rhizosphere soil of pulses and wheat grown in the semiarid prairie, in the context of climate change. • Can be used to compare similar systems in Australia, Ukraine, or elsewhere. • Of interest to researchers in the field of soil microbial ecology, agronomy, agroecology, and climate change.

## 1 Data

The data are from a 72-plot field experiment testing the influence of pulse crop frequency on root and rhizosphere bacteria, which was conducted in the semiarid prairie between 2010 and 2014; the data are from 2012 to 2014. They consist of sequences of amplicons of the V6–V8 regions of bacterial 16S rDNA from soil and root extracts obtained by pyrosequencing (Table 1), and levels of soil organic carbon, total carbon, pH, electrical conductivity, and extractible sulfate sulfur, copper, iron, manganese, zinc, potassium, mineral nitrogen (NH_4_-N and NO_3_-N), phosphate phosphorus, calcium, and magnesium in the 0–15 cm soil layer and mineral nitrogen and phosphate in the 0–120 cm soil layer (Table 2). Treatments impacted the yield of the wheat crop grown in the last (4th) phase of the rotations. This wheat grain yield data are also given below (Table 3).

**Table 1:** Sequence database deposited in NCBI.

NCBI reference				Experimental sample identification						
Accession	Organism	Tax ID	Bio Project	Year	Exp. cycle	Plot	Crop	Rotation	Block	Sample_Type
SAMN08053356	rhizosphere metagenome	410658	PRJNA419421	2012	1	1	PEA	P-W-P-W	1	rhizosphere
SAMN08053357	rhizosphere metagenome	410658	PRJNA419421	2012	1	2	LEN	L-W-L-W	1	rhizosphere
SAMN08053358	rhizosphere metagenome	410658	PRJNA419421	2012	1	3	WHT	C-W-W-W	1	rhizosphere
SAMN08053359	rhizosphere metagenome	410658	PRJNA419421	2012	1	6	WHT	W-W-W-W	1	rhizosphere
SAMN08053360	rhizosphere metagenome	410658	PRJNA419421	2012	1	8	WHT	P-W-W-W	1	rhizosphere
SAMN08053361	rhizosphere metagenome	410658	PRJNA419421	2012	1	9	CHP	C-C-C-W	1	rhizosphere
SAMN08053362	rhizosphere metagenome	410658	PRJNA419421	2012	1	10	CHP	C-W-C-W	1	rhizosphere
SAMN08053363	rhizosphere metagenome	410658	PRJNA419421	2012	1	13	LEN	L-L-L-W	1	rhizosphere
SAMN08053364	rhizosphere metagenome	410658	PRJNA419421	2012	1	14	PEA	P-P-P-W	1	rhizosphere
SAMN08053365	rhizosphere metagenome	410658	PRJNA419421	2012	1	15	WHT	P-W-W-W	2	rhizosphere
SAMN08053366	rhizosphere metagenome	410658	PRJNA419421	2012	1	17	CHP	C-W-C-W	2	rhizosphere
SAMN08053367	rhizosphere metagenome	410658	PRJNA419421	2012	1	19	PEA	P-W-P-W	2	rhizosphere
SAMN08053368	rhizosphere metagenome	410658	PRJNA419421	2012	1	20	PEA	P-P-P-W	2	rhizosphere
SAMN08053369	rhizosphere metagenome	410658	PRJNA419421	2012	1	21	LEN	L-W-L-W	2	rhizosphere
SAMN08053370	rhizosphere metagenome	410658	PRJNA419421	2012	1	22	CHP	C-C-C-W	2	rhizosphere
SAMN08053371	rhizosphere metagenome	410658	PRJNA419421	2012	1	23	WHT	C-W-W-W	2	rhizosphere
SAMN08053372	rhizosphere metagenome	410658	PRJNA419421	2012	1	24	LEN	L-L-L-W	2	rhizosphere
SAMN08053373	rhizosphere metagenome	410658	PRJNA419421	2012	1	28	WHT	W-W-W-W	2	rhizosphere
SAMN08053374	rhizosphere metagenome	410658	PRJNA419421	2012	1	29	CHP	C-W-C-W	3	rhizosphere
SAMN08053375	rhizosphere metagenome	410658	PRJNA419421	2012	1	30	PEA	P-W-P-W	3	rhizosphere
SAMN08053376	rhizosphere metagenome	410658	PRJNA419421	2012	1	31	LEN	L-W-L-W	3	rhizosphere
SAMN08053377	rhizosphere metagenome	410658	PRJNA419421	2012	1	32	PEA	P-P-P-W	3	rhizosphere
SAMN08053378	rhizosphere metagenome	410658	PRJNA419421	2012	1	35	WHT	C-W-W-W	3	rhizosphere
SAMN08053379	rhizosphere metagenome	410658	PRJNA419421	2012	1	36	LEN	L-L-L-W	3	rhizosphere
SAMN08053380	rhizosphere metagenome	410658	PRJNA419421	2012	1	38	WHT	W-W-W-W	3	rhizosphere
SAMN08053381	rhizosphere metagenome	410658	PRJNA419421	2012	1	40	CHP	C-C-C-W	3	rhizosphere
SAMN08053382	rhizosphere metagenome	410658	PRJNA419421	2012	1	41	WHT	P-W-W-W	3	rhizosphere
SAMN08053383	rhizosphere metagenome	410658	PRJNA419421	2012	1	43	WHT	W-W-W-W	4	rhizosphere
SAMN08053384	rhizosphere metagenome	410658	PRJNA419421	2012	1	45	CHP	C-C-C-W	4	rhizosphere
SAMN08053385	rhizosphere metagenome	410658	PRJNA419421	2012	1	46	WHT	C-W-W-W	4	rhizosphere
SAMN08053386	rhizosphere metagenome	410658	PRJNA419421	2012	1	47	LEN	L-L-L-W	4	rhizosphere
SAMN08053387	rhizosphere metagenome	410658	PRJNA419421	2012	1	48	WHT	P-W-W-W	4	rhizosphere
SAMN08053388	rhizosphere metagenome	410658	PRJNA419421	2012	1	49	PEA	P-P-P-W	4	rhizosphere
SAMN08053389	rhizosphere metagenome	410658	PRJNA419421	2012	1	52	PEA	P-W-P-W	4	rhizosphere
SAMN08053390	rhizosphere metagenome	410658	PRJNA419421	2012	1	53	CHP	C-W-C-W	4	rhizosphere
SAMN08053391	rhizosphere metagenome	410658	PRJNA419421	2012	1	55	LEN	L-W-L-W	4	rhizosphere
SAMN08053392	root metagenome	1118232	PRJNA419421	2013	1	1	WHT	P-W-P-W	1	root
SAMN08053393	root metagenome	1118232	PRJNA419421	2013	1	2	WHT	L-W-L-W	1	root
SAMN08053394	root metagenome	1118232	PRJNA419421	2013	1	3	WHT	C-W-W-W	1	root
SAMN08053395	root metagenome	1118232	PRJNA419421	2013	1	6	WHT	W-W-W-W	1	root
SAMN08053396	root metagenome	1118232	PRJNA419421	2013	1	8	WHT	P-W-W-W	1	root
SAMN08053397	root metagenome	1118232	PRJNA419421	2013	1	9	WHT	C-C-C-W	1	root
SAMN08053398	root metagenome	1118232	PRJNA419421	2013	1	10	WHT	C-W-C-W	1	root
SAMN08053399	root metagenome	1118232	PRJNA419421	2013	1	13	WHT	L-L-L-W	1	root
SAMN08053400	root metagenome	1118232	PRJNA419421	2013	1	14	WHT	P-P-P-W	1	root
SAMN08053401	root metagenome	1118232	PRJNA419421	2013	1	15	WHT	P-W-W-W	2	root
SAMN08053402	root metagenome	1118232	PRJNA419421	2013	1	17	WHT	C-W-C-W	2	root
SAMN08053403	root metagenome	1118232	PRJNA419421	2013	1	19	WHT	P-W-P-W	2	root
SAMN08053404	root metagenome	1118232	PRJNA419421	2013	1	20	WHT	P-P-P-W	2	root
SAMN08053405	root metagenome	1118232	PRJNA419421	2013	1	21	WHT	L-W-L-W	2	root
SAMN08053406	root metagenome	1118232	PRJNA419421	2013	1	22	WHT	C-C-C-W	2	root
SAMN08053407	root metagenome	1118232	PRJNA419421	2013	1	23	WHT	C-W-W-W	2	root
SAMN08053408	root metagenome	1118232	PRJNA419421	2013	1	24	WHT	L-L-L-W	2	root
SAMN08053409	root metagenome	1118232	PRJNA419421	2013	1	28	WHT	W-W-W-W	2	root
SAMN08053410	root metagenome	1118232	PRJNA419421	2013	1	29	WHT	C-W-C-W	3	root
SAMN08053411	root metagenome	1118232	PRJNA419421	2013	1	30	WHT	P-W-P-W	3	root
SAMN08053412	root metagenome	1118232	PRJNA419421	2013	1	31	WHT	L-W-L-W	3	root
SAMN08053413	root metagenome	1118232	PRJNA419421	2013	1	32	WHT	P-P-P-W	3	root
SAMN08053414	root metagenome	1118232	PRJNA419421	2013	1	35	WHT	C-W-W-W	3	root
SAMN08053415	root metagenome	1118232	PRJNA419421	2013	1	36	WHT	L-L-L-W	3	root
SAMN08053416	root metagenome	1118232	PRJNA419421	2013	1	38	WHT	W-W-W-W	3	root
SAMN08053417	root metagenome	1118232	PRJNA419421	2013	1	40	WHT	C-C-C-W	3	root
SAMN08053418	root metagenome	1118232	PRJNA419421	2013	1	41	WHT	P-W-W-W	3	root
SAMN08053419	root metagenome	1118232	PRJNA419421	2013	1	43	WHT	W-W-W-W	4	root
SAMN08053420	root metagenome	1118232	PRJNA419421	2013	1	45	WHT	C-C-C-W	4	root
SAMN08053421	root metagenome	1118232	PRJNA419421	2013	1	46	WHT	C-W-W-W	4	root
SAMN08053422	root metagenome	1118232	PRJNA419421	2013	1	47	WHT	L-L-L-W	4	root
SAMN08053423	root metagenome	1118232	PRJNA419421	2013	1	48	WHT	P-W-W-W	4	root
SAMN08053424	root metagenome	1118232	PRJNA419421	2013	1	49	WHT	P-P-P-W	4	root
SAMN08053425	root metagenome	1118232	PRJNA419421	2013	1	52	WHT	P-W-P-W	4	root
SAMN08053426	root metagenome	1118232	PRJNA419421	2013	1	53	WHT	C-W-C-W	4	root
SAMN08053427	root metagenome	1118232	PRJNA419421	2013	1	55	WHT	L-W-L-W	4	root
SAMN08053428	rhizosphere metagenome	410658	PRJNA419421	2013	2	1	CHP	C-W-C-W	1	rhizosphere
SAMN08053429	rhizosphere metagenome	410658	PRJNA419421	2013	2	2	PEA	P-W-P-W	1	rhizosphere
SAMN08053430	rhizosphere metagenome	410658	PRJNA419421	2013	2	3	LEN	L-W-L-W	1	rhizosphere
SAMN08053431	rhizosphere metagenome	410658	PRJNA419421	2013	2	4	PEA	P-P-P-W	1	rhizosphere
SAMN08053432	rhizosphere metagenome	410658	PRJNA419421	2013	2	5	LEN	L-L-L-W	2	rhizosphere
SAMN08053433	rhizosphere metagenome	410658	PRJNA419421	2013	2	7	WHT	C-W-W-W	1	rhizosphere
SAMN08053434	rhizosphere metagenome	410658	PRJNA419421	2013	2	8	LEN	L-L-L-W	1	rhizosphere
SAMN08053435	rhizosphere metagenome	410658	PRJNA419421	2013	2	10	WHT	W-W-W-W	1	rhizosphere
SAMN08053436	rhizosphere metagenome	410658	PRJNA419421	2013	2	12	CHP	C-C-C-W	1	rhizosphere
SAMN08053437	rhizosphere metagenome	410658	PRJNA419421	2013	2	13	WHT	P-W-W-W	1	rhizosphere
SAMN08053438	rhizosphere metagenome	410658	PRJNA419421	2013	2	15	WHT	W-W-W-W	2	rhizosphere
SAMN08053439	rhizosphere metagenome	410658	PRJNA419421	2013	2	18	WHT	C-W-W-W	2	rhizosphere
SAMN08053440	rhizosphere metagenome	410658	PRJNA419421	2013	2	20	WHT	P-W-W-W	2	rhizosphere
SAMN08053441	rhizosphere metagenome	410658	PRJNA419421	2013	2	21	PEA	P-P-P-W	2	rhizosphere
SAMN08053442	rhizosphere metagenome	410658	PRJNA419421	2013	2	24	PEA	P-W-P-W	2	rhizosphere
SAMN08053443	rhizosphere metagenome	410658	PRJNA419421	2013	2	25	CHP	C-W-C-W	2	rhizosphere
SAMN08053444	rhizosphere metagenome	410658	PRJNA419421	2013	2	27	LEN	L-W-L-W	2	rhizosphere
SAMN08053445	rhizosphere metagenome	410658	PRJNA419421	2013	2	29	PEA	P-W-P-W	3	rhizosphere
SAMN08053446	rhizosphere metagenome	410658	PRJNA419421	2013	2	30	LEN	L-W-L-W	3	rhizosphere
SAMN08053447	rhizosphere metagenome	410658	PRJNA419421	2013	2	31	WHT	C-W-W-W	3	rhizosphere
SAMN08053448	rhizosphere metagenome	410658	PRJNA419421	2013	2	34	WHT	W-W-W-W	3	rhizosphere
SAMN08053449	rhizosphere metagenome	410658	PRJNA419421	2013	2	36	WHT	P-W-W-W	3	rhizosphere
SAMN08053450	rhizosphere metagenome	410658	PRJNA419421	2013	2	37	CHP	C-C-C-W	3	rhizosphere
SAMN08053451	rhizosphere metagenome	410658	PRJNA419421	2013	2	38	CHP	C-W-C-W	3	rhizosphere
SAMN08053452	rhizosphere metagenome	410658	PRJNA419421	2013	2	41	LEN	L-L-L-W	3	rhizosphere
SAMN08053453	rhizosphere metagenome	410658	PRJNA419421	2013	2	42	PEA	P-P-P-W	3	rhizosphere
SAMN08053454	rhizosphere metagenome	410658	PRJNA419421	2013	2	43	WHT	P-W-W-W	4	rhizosphere
SAMN08053455	rhizosphere metagenome	410658	PRJNA419421	2013	2	45	CHP	C-W-C-W	4	rhizosphere
SAMN08053456	rhizosphere metagenome	410658	PRJNA419421	2013	2	47	PEA	P-W-P-W	4	rhizosphere
SAMN08053457	rhizosphere metagenome	410658	PRJNA419421	2013	2	48	PEA	P-P-P-W	4	rhizosphere
SAMN08053458	rhizosphere metagenome	410658	PRJNA419421	2013	2	49	LEN	L-W-L-W	4	rhizosphere
SAMN08053459	rhizosphere metagenome	410658	PRJNA419421	2013	2	50	CHP	C-C-C-W	4	rhizosphere
SAMN08053460	rhizosphere metagenome	410658	PRJNA419421	2013	2	51	WHT	C-W-W-W	4	rhizosphere
SAMN08053461	rhizosphere metagenome	410658	PRJNA419421	2013	2	52	LEN	L-L-L-W	4	rhizosphere
SAMN08053462	rhizosphere metagenome	410658	PRJNA419421	2013	2	56	WHT	W-W-W-W	4	rhizosphere
SAMN08053463	root metagenome	1118232	PRJNA419421	2014	2	1	WHT	C-W-C-W	1	root
SAMN08053464	root metagenome	1118232	PRJNA419421	2014	2	2	WHT	P-W-P-W	1	root
SAMN08053465	root metagenome	1118232	PRJNA419421	2014	2	3	WHT	L-W-L-W	1	root
SAMN08053466	root metagenome	1118232	PRJNA419421	2014	2	4	WHT	P-P-P-W	1	root
SAMN08053467	root metagenome	1118232	PRJNA419421	2014	2	5	WHT	L-L-L-W	2	root
SAMN08053468	root metagenome	1118232	PRJNA419421	2014	2	7	WHT	C-W-W-W	1	root
SAMN08053469	root metagenome	1118232	PRJNA419421	2014	2	8	WHT	L-L-L-W	1	root
SAMN08053470	root metagenome	1118232	PRJNA419421	2014	2	10	WHT	W-W-W-W	1	root
SAMN08053471	root metagenome	1118232	PRJNA419421	2014	2	12	WHT	C-C-C-W	1	root
SAMN08053472	root metagenome	1118232	PRJNA419421	2014	2	13	WHT	P-W-W-W	1	root
SAMN08053473	root metagenome	1118232	PRJNA419421	2014	2	15	WHT	W-W-W-W	2	root
SAMN08053474	root metagenome	1118232	PRJNA419421	2014	2	17	WHT	C-C-C-W	2	root
SAMN08053475	root metagenome	1118232	PRJNA419421	2014	2	18	WHT	C-W-W-W	2	root
SAMN08053476	root metagenome	1118232	PRJNA419421	2014	2	20	WHT	P-W-W-W	2	root
SAMN08053477	root metagenome	1118232	PRJNA419421	2014	2	21	WHT	P-P-P-W	2	root
SAMN08053478	root metagenome	1118232	PRJNA419421	2014	2	24	WHT	P-W-P-W	2	root
SAMN08053479	root metagenome	1118232	PRJNA419421	2014	2	25	WHT	C-W-C-W	2	root
SAMN08053480	root metagenome	1118232	PRJNA419421	2014	2	27	WHT	L-W-L-W	2	root
SAMN08053481	root metagenome	1118232	PRJNA419421	2014	2	29	WHT	P-W-P-W	3	root
SAMN08053482	root metagenome	1118232	PRJNA419421	2014	2	30	WHT	L-W-L-W	3	root
SAMN08053483	root metagenome	1118232	PRJNA419421	2014	2	31	WHT	C-W-W-W	3	root
SAMN08053484	root metagenome	1118232	PRJNA419421	2014	2	34	WHT	W-W-W-W	3	root
SAMN08053485	root metagenome	1118232	PRJNA419421	2014	2	36	WHT	P-W-W-W	3	root
SAMN08053486	root metagenome	1118232	PRJNA419421	2014	2	37	WHT	C-C-C-W	3	root
SAMN08053487	root metagenome	1118232	PRJNA419421	2014	2	38	WHT	C-W-C-W	3	root
SAMN08053488	root metagenome	1118232	PRJNA419421	2014	2	41	WHT	L-L-L-W	3	root
SAMN08053489	root metagenome	1118232	PRJNA419421	2014	2	42	WHT	P-P-P-W	3	root
SAMN08053490	root metagenome	1118232	PRJNA419421	2014	2	43	WHT	P-W-W-W	4	root
SAMN08053491	root metagenome	1118232	PRJNA419421	2014	2	45	WHT	C-W-C-W	4	root
SAMN08053492	root metagenome	1118232	PRJNA419421	2014	2	47	WHT	P-W-P-W	4	root
SAMN08053493	root metagenome	1118232	PRJNA419421	2014	2	48	WHT	P-P-P-W	4	root
SAMN08053494	root metagenome	1118232	PRJNA419421	2014	2	49	WHT	L-W-L-W	4	root
SAMN08053495	root metagenome	1118232	PRJNA419421	2014	2	50	WHT	C-C-C-W	4	root
SAMN08053496	root metagenome	1118232	PRJNA419421	2014	2	51	WHT	C-W-W-W	4	root
SAMN08053497	root metagenome	1118232	PRJNA419421	2014	2	52	WHT	L-L-L-W	4	root
SAMN08053498	root metagenome	1118232	PRJNA419421	2014	2	56	WHT	W-W-W-W	4	root

**Table 2:** Soil properties (0–15 cm) in the fall of rotation phase-3 in 2012 and 2013 for cycles 1 and 2, respectively.

Cycle	Plot	Rep	Rotation	Total C (%)	Organic C (%)	Mineral N in soil profile^a^ (mg kg^−1^)	Phosphate in soil profile^a^ (mg kg^−1^)	pH	Electrical conductivity (mS)	SO_4_-S	Cu	Fe	Mn	Zn	K	Mineral N	PO^4^-P	Ca	Mg
										(mg kg-^1^)									
1	1	1	P-W-P-W	16.5	16.5	90.7	70.40	6.51	0.70	4.83	1.33	23.90	18.50	0.96	433.07	3.03	36.89	1670.00	460.40
1	2	1	L-W-L-W	19.4	16.8	17.6	33.10	6.46	0.72	6.53	1.12	30.16	36.63	1.23	484.25	3.48	38.82	1561.87	438.72
1	3	1	C-W-W-W	19.1	17.1	18.5	46.40	6.50	0.54	5.07	0.77	30.66	16.94	1.12	587.30	5.04	41.88	1510.97	387.92
1	6	1	W-W-W-W	16.0	14.5	29.3	30.10	6.26	0.37	3.31	1.37	34.68	13.41	1.22	574.30	2.69	44.52	1467.81	414.79
1	8	1	P-W-W-W	16.7	15.1	31.6	40.90	6.15	0.36	3.99	0.90	44.74	21.11	1.01	561.00	3.66	44.08	1334.32	394.74
1	9	1	C-C-C-W	14.6	14.6	44.6	28.30	6.38	0.73	5.52	1.08	26.72	37.83	1.06	455.00	18.66	47.00	1621.74	442.66
1	10	1	C-W-C-W	13.9	13.5	28.0	42.60	6.77	0.77	3.93	0.99	6.07	20.38	0.70	343.87	4.33	37.02	1968.82	496.18
1	13	1	L-L-L-W	15.1	13.6	27.0	35.90	6.35	0.82	5.59	1.06	16.57	25.06	1.06	338.06	3.79	35.59	1744.95	506.85
1	14	1	P-P-P-W	14.6	14.5	59.7	45.60	6.31	0.79	4.54	1.34	16.53	15.67	0.87	465.86	6.41	50.38	1826.31	473.30
1	15	2	P-W-W-W	17.8	17.8	16.8	26.90	6.18	0.36	3.17	1.04	36.52	12.65	1.02	462.30	2.90	39.76	1550.89	412.67
1	17	2	C-W-C-W	17.7	16.1	22.7	86.40	6.13	0.51	4.39	1.17	42.13	23.70	1.03	551.18	2.99	43.27	1494.04	400.70
1	19	2	P-W-P-W	23.0	16.9	94.9	48.80	6.69	0.95	5.32	0.92	13.68	22.36	0.93	465.14	6.43	37.09	1755.92	465.41
1	20	2	P-P-P-W	19.5	16.3	178.4	38.80	7.07	1.10	5.31	0.91	5.34	15.03	0.83	442.46	7.78	38.95	1971.77	467.74
1	21	2	L-W-L-W	16.8	16.2	18.7	46.60	6.93	0.95	5.19	1.45	5.26	18.05	0.82	395.26	2.31	37.96	1897.62	460.84
1	22	2	C-C-C-W	18.9	17.1	36.3	43.60	6.41	0.94	5.75	1.20	24.74	35.63	1.10	451.19	10.62	51.35	1651.92	460.26
1	23	2	C-W-W-W	16.5	16.1	35.7	58.10	6.08	0.39	3.38	1.51	49.66	23.00	1.14	455.36	3.51	39.62	1359.71	402.30
1	24	2	L-L-L-W	17.7	14.3	29.5	71.90	6.29	0.60	6.15	1.41	35.50	28.05	1.15	422.55	5.65	37.12	1461.30	410.51
1	28	2	W-W-W-W	19.0	16.2	22.1	99.00	6.02	0.35	2.94	0.92	47.97	22.73	1.09	465.95	3.81	40.53	1388.67	388.57
1	29	3	C-W-C-W	18.3	17.2	38.2	90.60	6.78	1.01	5.34	0.90	5.09	15.96	0.76	514.82	4.76	34.92	1956.75	491.45
1	30	3	P-W-P-W	18.8	15.7	98.3	47.30	7.30	0.76	4.30	0.93	1.65	5.69	0.75	475.81	6.71	25.44	2290.91	451.01
1	31	3	L-W-L-W	19.0	19.0	23.6	36.50	6.73	0.89	5.09	1.71	8.71	19.96	1.03	545.27	5.91	40.94	1844.18	456.80
1	32	3	P-P-P-W	18.3	17.0	110.8	57.60	6.39	0.56	4.39	0.99	39.27	29.05	0.99	552.32	7.00	40.72	1520.15	416.03
1	35	3	C-W-W-W	17.3	16.2	51.2	38.20	6.02	0.35	4.28	2.37	30.13	21.35	1.27	454.82	3.29	41.85	1560.37	431.49
1	36	3	L-L-L-W	20.5	18.4	23.2	68.40	6.48	0.80	4.93	1.85	22.83	24.66	1.26	508.03	7.03	45.90	1672.00	443.30
1	38	3	W-W-W-W	17.0	16.6	13.8	22.90	5.93	0.33	2.94	1.78	60.07	21.23	1.40	518.00	3.16	38.90	1347.39	347.59
1	40	3	C-C-C-W	20.8	17.0	0.0	80.40	6.05	0.72	4.85	1.28	43.06	41.68	1.13	471.46	5.14	41.42	1461.85	375.20
1	41	3	P-W-W-W	16.0	15.0	32.5	29.80	6.07	0.26	2.04	1.74	36.16	14.70	0.97	406.62	2.41	32.92	1440.29	363.87
1	43	4	W-W-W-W	15.8	14.6	21.7	30.70	6.79	0.48	3.17	1.42	4.91	10.47	0.73	389.65	3.26	31.50	1897.20	477.84
1	45	4	C-C-C-W	21.4	17.4	45.9	19.20	6.24	0.88	7.62	1.50	34.52	36.22	1.18	616.07	6.59	41.13	1433.53	427.68
1	46	4	C-W-W-W	23.0	22.0	31.9	57.30	6.02	0.23	3.22	3.31	64.15	25.50	2.60	442.16	5.82	40.24	1311.00	389.90
1	47	4	L-L-L-W	16.1	15.6	31.0	56.10	5.96	0.28	3.30	3.03	55.29	23.98	1.39	390.32	15.06	43.22	1381.94	410.61
1	48	4	P-W-W-W	15.4	15.4	32.9	41.20	6.19	0.37	3.19	1.14	43.63	22.71	1.24	478.57	6.00	40.22	1426.14	412.17
1	49	4	P-P-P-W	18.3	16.2	146.1	87.10	6.78	0.63	3.67	0.73	14.56	15.96	0.87	477.09	11.37	42.93	1796.79	456.74
1	52	4	P-W-P-W	15.7	15.7	48.4	90.90	5.97	0.27	2.41	1.05	63.54	27.39	1.20	386.09	6.03	38.55	1439.64	345.68
1	53	4	C-W-C-W	16.8	16.8	34.2	49.50	5.92	0.31	2.66	0.92	69.20	22.78	1.05	369.05	3.67	41.88	1402.59	358.15
1	55	4	L-W-L-W	15.4	15.3	29.7	35.60	6.14	0.58	3.57	0.89	43.17	29.04	0.98	417.00	1.74	32.73	1470.89	387.35
2	1	1	C-W-C-W	17.3	17.2	131.2	83.70	5.98	0.32	4.69	0.79	50.10	20.64	1.18	553.64	2.63	44.98	1567.00	359.50
2	2	1	P-W-P-W	15.8	15.5	37.8	19.50	6.06	0.27	2.54	2.01	49.22	17.55	1.26	464.84	7.46	44.96	1424.55	352.11
2	3	1	L-W-L-W	16.4	16.4	48.6	70.20	6.02	0.49	3.95	1.27	45.44	21.58	1.31	585.34	4.48	46.73	1486.93	362.17
2	4	1	P-P-P-W	17.9	16.5	111.3	32.50	6.17	0.42	4.22	0.54	45.63	18.35	1.10	645.00	8.26	37.38	1404.39	325.50
2	5	2	L-L-L-W	15.6	15.0	118.9	16.30	5.89	0.28	3.37	0.72	50.72	22.03	1.06	520.16	10.32	46.69	1388.89	325.05
2	7	1	C-W-W-W	17.9	17.0	47.1	62.00	6.23	0.38	4.98	0.73	46.81	14.91	1.44	609.13	2.28	42.96	1432.53	346.63
2	8	1	L-L-L-W	17.8	16.8	79.9	73.90	5.90	0.33	4.54	0.81	51.94	17.63	1.06	421.26	10.37	45.45	1455.91	342.08
2	10	1	W-W-W-W	17.8	17.8	47.4	69.80	6.34	0.45	5.76	0.95	33.10	15.43	1.45	504.98	2.53	33.37	1720.33	397.39
2	12	1	C-C-C-W	17.0	16.6	94.5	37.30	6.02	0.46	3.24	1.12	46.04	16.80	0.99	614.09	1.90	42.34	1499.99	366.57
2	13	1	P-W-W-W	18.5	18.1	56.7	61.00	6.35	0.36	4.54	0.65	33.29	13.93	1.10	657.00	4.76	44.58	1580.20	363.07
2	15	2	W-W-W-W	17.4	17.3	56.7	96.30	6.31	0.39	4.77	0.95	30.48	15.68	1.26	835.66	2.35	53.01	1482.11	347.12
2	17	2	C-C-C-W	.	.	.	.	.	.	.	.	.	.	.	.	.	.	.	.
2	18	2	C-W-W-W	16.3	16.3	61.2	36.10	6.69	0.52	5.84	1.25	18.60	9.94	0.95	868.17	2.05	43.59	1721.55	372.35
2	20	2	P-W-W-W	15.4	15.4	73.7	45.10	5.92	0.25	7.22	0.73	53.30	17.61	0.95	658.10	3.20	44.45	1392.85	335.91
2	21	2	P-P-P-W	16.5	16.5	96.0	42.40	5.78	0.23	6.39	1.29	52.86	18.42	1.06	441.93	11.97	55.49	1431.73	346.29
2	24	2	P-W-P-W	17.6	17.6	105.1	54.50	5.91	0.36	4.42	1.43	57.79	21.86	1.25	501.01	7.77	45.83	1494.03	344.32
2	25	2	C-W-C-W	18.9	18.4	79.9	63.50	6.12	0.52	5.21	0.89	52.20	24.86	1.30	667.67	3.39	43.96	1448.89	343.29
2	27	2	L-W-L-W	17.1	17.1	104.1	50.90	6.40	0.73	6.39	1.50	36.08	18.27	1.26	779.88	7.05	40.26	1588.06	385.33
2	29	3	P-W-P-W	17.6	17.2	58.6	43.80	6.39	0.54	5.87	0.62	37.31	17.06	1.01	714.14	7.27	46.24	1447.53	382.77
2	30	3	L-W-L-W	17.5	17.4	52.9	5.60	6.45	0.72	5.84	0.71	25.86	16.64	0.98	687.50	4.03	37.24	1614.69	388.03
2	31	3	C-W-W-W	17.4	17.4	73.7	70.90	6.70	0.44	7.17	0.66	23.26	10.82	0.97	739.21	3.14	43.22	1721.22	405.36
2	34	3	W-W-W-W	16.2	15.9	60.9	34.70	5.98	0.27	3.68	0.76	53.32	15.74	1.07	759.96	3.55	42.03	1102.79	286.03
2	36	3	P-W-W-W	16.3	16.3	55.9	90.60	5.65	0.21	3.71	1.14	58.89	16.87	0.94	501.98	4.23	41.75	1360.45	358.84
2	37	3	C-C-C-W	20.6	19.1	9.3	45.60	5.90	0.32	3.56	1.21	57.74	27.59	2.79	470.35	5.77	52.63	1522.04	387.47
2	38	3	C-W-C-W	18.2	17.9	75.8	61.50	5.88	0.33	4.07	1.16	56.21	24.52	1.28	538.15	5.00	43.45	1399.41	356.87
2	41	3	L-L-L-W	22.4	19.9	49.0	73.10	6.55	0.68	5.69	0.63	23.33	16.80	0.81	322.71	4.76	36.41	1926.18	425.00
2	42	3	P-P-P-W	16.7	16.7	124.2	58.30	6.85	0.51	3.99	2.30	16.27	11.13	0.94	421.00	9.56	29.80	2585.00	388.80
2	43	4	P-W-W-W	16.4	16.4	69.4	37.80	6.07	0.35	3.75	0.70	49.24	17.06	1.18	544.64	2.10	33.35	1428.00	377.40
2	45	4	C-W-C-W	18.2	17.8	60.7	53.30	6.03	0.42	3.70	1.14	50.58	20.52	1.05	475.00	2.28	36.38	1413.82	382.76
2	47	4	P-W-P-W	18.2	15.5	123.8	34.60	5.99	0.42	4.08	0.99	53.59	20.61	1.03	462.21	7.21	29.77	1395.83	354.76
2	48	4	P-P-P-W	22.4	20.5	129.8	45.50	5.76	0.32	3.36	2.22	65.16	28.98	1.26	420.64	8.25	54.94	1349.69	335.17
2	49	4	L-W-L-W	17.5	16.0	101.5	28.00	5.93	0.62	5.72	0.79	51.93	25.48	1.11	483.14	3.19	43.15	1335.31	344.24
2	50	4	C-C-C-W	15.7	15.7	63.1	21.90	5.62	0.23	2.58	1.43	55.66	21.52	0.90	293.00	2.64	34.86	1414.17	372.65
2	51	4	C-W-W-W	16.0	16.0	65.2	27.70	5.82	0.33	5.11	0.95	43.56	14.28	0.97	601.00	2.20	30.68	1402.00	378.90
2	52	4	L-L-L-W	14.8	14.8	75.4	10.40	5.50	0.02	2.00	1.43	57.37	24.12	0.83	321.86	3.87	41.07	1327.65	338.58
2	56	4	W-W-W-W	21.2	19.8	32.7	26.80	6.89	0.78	5.69	0.97	3.74	5.11	0.78	456.48	2.04	29.90	2106.78	477.54

a The soil profile is the top 120 cm soil layer.

**Table 3:** Grain yield of phase-4 wheat in 2013 and 2014, for cycles 1 and 2, respectively.

Cycle	Plot	Rep	Rotation	Grain yield (kg ha^−1^)
1	1	1	P-W-P-W	2704.3
1	2	1	L-W-L-W	2478.9
1	3	1	C-W-W-W	2365.6
1	6	1	W-W-W-W	1706.5
1	8	1	P-W-W-W	2047.2
1	9	1	C-C-C-W	2792.5
1	10	1	C-W-C-W	2107.6
1	13	1	L-L-L-W	2238.6
1	14	1	P-P-P-W	2796.6
1	15	2	P-W-W-W	2406.3
1	17	2	C-W-C-W	2529.2
1	19	2	P-W-P-W	2714.5
1	20	2	P-P-P-W	2923.5
1	21	2	L-W-L-W	2794.6
1	22	2	C-C-C-W	2293.0
1	23	2	C-W-W-W	2123.3
1	24	2	L-L-L-W	2512.9
1	28	2	W-W-W-W	2058.1
1	29	3	C-W-C-W	2151.1
1	30	3	P-W-P-W	2729.4
1	31	3	L-W-L-W	2954.1
1	32	3	P-P-P-W	3233.8
1	35	3	C-W-W-W	2561.1
1	36	3	L-L-L-W	2591.6
1	38	3	W-W-W-W	1970.5
1	40	3	C-C-C-W	2259.7
1	41	3	P-W-W-W	2347.9
1	43	4	W-W-W-W	1920.3
1	45	4	C-C-C-W	2227.1
1	46	4	C-W-W-W	2290.2
1	47	4	L-L-L-W	2685.3
1	48	4	P-W-W-W	2124.6
1	49	4	P-P-P-W	3055.9
1	52	4	P-W-P-W	2459.3
1	53	4	C-W-C-W	2544.1
1	55	4	L-W-L-W	2510.8
2	1	1	C-W-C-W	2713.1
2	2	1	P-W-P-W	2910.0
2	3	1	L-W-L-W	2695.5
2	4	1	P-P-P-W	3235.1
2	5	2	L-L-L-W	3142.8
2	7	1	C-W-W-W	2417.8
2	8	1	L-L-L-W	2872.0
2	10	1	W-W-W-W	2293.0
2	12	1	C-C-C-W	2154.5
2	13	1	P-W-W-W	2326.9
2	15	2	W-W-W-W	2510.8
2	17	2	C-C-C-W	2702.3
2	18	2	C-W-W-W	2472.2
2	20	2	P-W-W-W	2504.1
2	21	2	P-P-P-W	3400.7
2	24	2	P-W-P-W	2806.1
2	25	2	C-W-C-W	2436.2
2	27	2	L-W-L-W	2605.2
2	29	3	P-W-P-W	2886.9
2	30	3	L-W-L-W	3205.2
2	31	3	C-W-W-W	2486.4
2	34	3	W-W-W-W	2605.9
2	36	3	P-W-W-W	2333.7
2	37	3	C-C-C-W	2358.1
2	38	3	C-W-C-W	2132.1
2	41	3	L-L-L-W	3043.0
2	42	3	P-P-P-W	3715.0
2	43	4	P-W-W-W	2376.4
2	45	4	C-W-C-W	2183.0
2	47	4	P-W-P-W	3003.0
2	48	4	P-P-P-W	3313.2
2	49	4	L-W-L-W	2732.8
2	50	4	C-C-C-W	2262.4
2	51	4	C-W-W-W	2093.4
2	52	4	L-L-L-W	2648.0
2	56	4	W-W-W-W	2412.4

## 2 Experimental design, materials, and methods

The 4-year crop rotation experiment was conducted from 2010 to 2013 (cycle-1), and repeated from 2011 to 2014 (cycle-2). The two cycles of the experiment were located side-by-side on the same land, at the Swift Current Research and Development Centre of Agriculture and Agri-Food Canada, in southwest Saskatchewan, Canada, a semiarid region. There were nine crop rotation treatments with pulse frequency ranging from zero to three. Treatments were: W-W-W-W, P-W-W-W, C-W-W-W, P-W-P-W, L-W-L-W, C-W-C-W, P-P-P-W, L-L-L-W, and C-C-C-W, where W = wheat, C = chickpea, P = pea, and L = lentil. In each of the two identical experimental cycles, treatments were randomized in four complete blocks. This experiment and the methodology employed are described in detail elsewhere [3].

Rhizosphere soil samples were taken in the fall following harvest of the third year crops (phase-3). Rhizosphere soil samples were collected from 3 to 4 root systems taken to a depth of approximately 30 cm with a shovel, from three randomly selected locations along the third and second rows in each plot. Plant roots were placed in Ziploc™ bags and kept in coolers on ice during sampling operation. Root samples were transported in a van over approximately 3 km and kept in a walk-in cold chamber at 4 °C until processing. Rhizosphere soil samples were immediately collected into Ziploc™ bags by brushing the soil remaining attached to roots after shaking, using a soft toothbrush. Rhizosphere soil samples were stored at −80 °C prior to molecular analysis.

Root sampling took place at the mid-bloom stage in the fourth year of the rotations. The roots of three plants randomly selected in each plot, were taken with a shovel to a depth of approximately 30 cm, and placed in Ziploc™ bags, in coolers on ice. Samples were momentarily placed in a walk-in cold chamber at 4 °C upon return to the crop research building. Roots were then washed in tap water and stored at −80 °C prior to molecular analysis.

Genomic DNA was extracted from 100 mg of fresh roots or 1 g of rhizosphere soil using DNeasy Plant Mini Kit (Qiagen) and Ultra Clean Soil DNA Isolation Kit (MoBio), respectively [3]. The V6–V8 regions of bacterial 16S rDNA were amplified using Nübel et al. (1996) [4] 968f/1401r primer set. The reverse primer (1401r) included the B adapter and the forward primer (968f), the A adapter and a 10-bp multiplex identifiers (1 of 12 different Roche MIDs). The PCR reaction volume was 20 μl and the reaction was conducted as described before [3]. Libraries were pooled, and purified using Agencourt AMPure XP (Beckman Coulter) and quality checked on an Agilent 2100 Bioanalyzer. Pools of 12 libraries in equimolar amounts were submitted to Genome Quebec for pyrosequencing with Roche GS FLX Titanium technology.

Sequences were trimmed, filtered, de-replicated, and clustered using the UPARSE pipeline [1] and deposited in NCBI (Table 1). Sequences were clustered into operational taxonomic units (OTUs) based on 97% similarity. The RDP classifier was then used to assign taxonomy to the OTUs using the 16S rRNA training set 16 [2].

The top (0–15 cm) soil layer of each plot was sampled in the fall of phase-3, after harvest. Duplicated samples were taken using a 30-mm-diameter soil corer. Samples were sieved through 2-mm mesh, and stored at 4 °C prior to analysis. Total carbon (C) was determined from a 12–15 mg soil sample on an Elemental analyzer (Elementar vario MICRO cube); 5–6 mg samples for organic carbon (C) were analyzed on the same equipment after pretreatment according to M. Baccanti and B. Colombo [5]. Soil extractions were prepared for soil mineral N [6], soil test P [7], potassium (K) [8] and soil extractible sulfur (S) [9]. Nitrogen, phosphorus and sulfur in these soil extracts were measured by colorimetry on the segmented flow auto-analyzer (Technicon, AAII System, Tarrytown, NY); K was measured by AAS on an atomic absorption spectrometer (Hitachi Z-8200). Copper (Cu), iron (Fe), manganese (Mn) and zinc (Zn) were extracted with diethylenetriaminepentaacetic acid/triethanolamine [10]; calcium (Ca) and magnesium (Mg) were extracted with ammonium acetate [11]; and all six were measured by ICP-OES (Fisher Scientific iCAP6300 Duo). Soil pH and electrical conductivity were measured using the saturated paste method [12] The N and phosphorus levels in the soil profile of each plot was also measured from duplicated 3-cm soil cores taken from the 0–120 cm top soil layer using the analytical procedures described above. The values presented in Table 2 are averages of the duplicates.

Phase-4 wheat grain yield (Table 3) was obtained by harvesting the central six rows of each plot using a plot combine, at harvest maturity.
