# Supplementary material for: Soil 16S DNA sequence data and corresponding soil property and wheat yield data from a 72-plot field experiment involving pulses and wheat crops grown in rotations in the semiarid prairie
Source: Data Brief. 2019 Feb 28;23:103790. doi: 10.1016/j.dib.2019.103790 (PMC6660571; doi:10.1016/j.dib.2019.103790)
Supplement: Multimedia component 1 [file mmc1.docx]

Authors have no conflict of interest to declare. Authors have no financial or personal interest, or belief that could affect their objectivity.
